# Dimensions of emotional intelligence related to physical and mental health and to health behaviors

**DOI:** 10.3389/fpsyg.2015.00317

**Published:** 2015-03-25

**Authors:** Enrique G. Fernández-Abascal, María Dolores Martín-Díaz

**Affiliations:** Department of Basic Psychology II, Faculty of Psychology, National University of Distance EducationMadrid, Spain

**Keywords:** trait emotional intelligence, mental health, physical health, health protective behavior, ager differences, gender differences

## Abstract

In this paper the relationship between emotional intelligence (EI) and health is examined. The current work investigated the dimensions of EI are sufficient to explain various components of physical and mental health, and various categories of health-related behaviors. A sample of 855 participants completed two measures of EI, the Trait Meta-Mood Scale and trait emotional intelligence questionnaire, a measure of health, the Health Survey SF-36 Questionnaire (SF-36); and a measure of health-related behaviors, the health behavior checklist. The results show that the EI dimensions analyzed are better predictors of mental health than of physical health. The EI dimensions that positively explain the Mental Health Component are Well-Being, Self-Control and Sociability, and negatively, Attention. Well-Being, Self-Control and Sociability positively explain the Physical Health Component. EI dimensions predict a lower percentage of health-related behaviors than they do health components. Emotionality and Repair predict the Preventive Health Behavior category, and only one dimension, Self-Control, predicts the Risk Taking Behavior category. Older people carry out more preventive behaviors for health.

## Introduction

Current studies suggest that higher emotional intelligence (EI) is linked to improved psychological and physical health, and a meta-analysis further emphasizes that the link between trait EI and mental health is important (Martins et al., 2010).

Emotional intelligence can be broadly defined as the ability to perceive, control, and evaluate emotions (Johnson et al., 2009). This set of characteristics, which deal with the perception, expression, and regulation of moods and emotions, suggests that there must be a direct link between EI and physical as well as psychological health (Tsaousis and Nikolaou, 2005).

There are different conceptualizations of EI in the research literature including: ability approaches, which examine relatively discrete mental abilities that process emotional information (Mayer et al., 2008); and trait approaches where trait EI is postulated to be a personality trait occupying the lower levels of the personality hierarchies (Petrides et al., 2007c). Peña-Sarrionandia et al. (2015) in a recent meta-analysis suggest that EI is a useful construct to capture individual differences in emotion regulation.

Emotional intelligence has been operationalized in different ways that can be divided into two general tendencies: maximum performance tests, which assess actual levels of EI performance (known as ability EI), and self-report questionnaires, which reflect typical EI functioning (known as trait EI or emotional self-efficacy; Pérez et al., 2005; Siegling et al., 2014).

Within the measures to assess trait EI, as indicated by Martins et al. (2010) in their comprehensive meta-analysis, two of the most frequently used are the Trait Meta-Mood Scale (TMMS; Salovey et al., 1995) and the trait emotional intelligence questionnaire (TEIQue; Petrides and Furnham, 2003a).

The TMMS is based on Salovey and Mayer’s (1990) EI model. This self-report measure evaluates three facets of the reflective processes that accompany mood states, termed the meta-mood experience (Salovey et al., 1995). The TMMS evaluates a “reasonable operationalization of aspects of EI” (Salovey et al., 1995, p. 147). This scale does not directly tap people’s emotional abilities but rather their perceived beliefs about their emotional abilities. Given its subjective nature, this instrument provides an index of what researchers have called a proxy for perceived EI (PEI; Salovey et al., 2002; Extremera and Fernández-Berrocal, 2005; Paek, 2006).

The TEIQue is based on Petrides’s model (Petrides and Furnham, 2001; Petrides et al., 2007c). Basically, this model, whose initial sampling domain was identified through content analysis of early EI and related models, aims at organizing in a single framework all affect-related aspects of personality. The construct seems to encompass variance of two kinds: a portion that is scattered across the higher-order dimensions of established personality taxonomies and a portion of variance that lies outside these dimensions (Petrides et al., 2007c). Trait EI is defined as a constellation of emotion-related self-perceptions and dispositions located at the lower levels of personality hierarchies (Petrides et al., 2007a).

Various works have studied the relation between EI and physical and mental health, emotional adjustment, psychological well-being, and life satisfaction (e.g., Goldman et al., 1996; Extremera and Fernández-Berrocal, 2002; Salovey et al., 2002; Saklofske et al., 2003; Petrides et al., 2007b; Johnson et al., 2009; Mavroveli et al., 2009; Andrei and Petrides, 2013; Costa et al., 2014; Laborde et al., 2014). However, as reported by Martins et al. (2010) in their meta-analysis, once the relation between EI and health is established, we need to focus on incremental validity issues. Another area of interest they point out is the relation between EI and specific types of health conditions. Some studies have also examined the relation between EI and consumption of substances such as alcohol and tobacco (e.g., Austin et al., 2005; Tsaousis and Nikolaou, 2005; Saklofske et al., 2007; Hill and Maggi, 2011), but the relation between EI, health behaviors, and addictions is still unclear (Kun and Demetrovics, 2010; Martins et al., 2010).

Health behaviors can be broadly defined as actions undertaken to maintain or improve health (Vickers et al., 1990). Research indicates that, rather than being independent, health behaviors occur in clusters or dimensions (Harris and Guten, 1979; Vickers et al., 1990). Summarizing the empirical patterns of association between behaviors, it has been shown that health behaviors tend to occur in combinations requiring between two and six dimensions or clusters (Harris and Guten, 1979; Tapp and Goldenthal, 1982; Krick and Sobal, 1990). When developing a measure to assess health behaviors, the health behavior checklist (HBC), Vickers et al. (1990), found that individual differences in health behavior can be conceptualized in terms of a hierarchical model. At the most general level, health behaviors formed two broad categories or dimensions, Preventive Health Behavior and Risk Taking Behavior.

### Present Study

The purpose of this investigation is to provide more evidence about the relationship of EI with physical and mental health, and with health behaviors.

The main objective of the work is to delimit the specific dimensions of EI that best predict various components of physical and mental health, and various categories of health-related behaviors.

For this purpose, we applied the two most frequently used measures of EI, which appraise different EI dimensions, thus including the greatest possible number of EI dimensions.

The investigations carried out have usually applied a single EI measure to verify the relation between this construct and health. In this paper we apply two measures of EI that assess different dimensions to obtain all possible dimensions of EI to determine the best physical and mental health.

Another objective is to determine if the dimensions of EI that predict better physical and mental health are the same for both types of health. We also intend to find out if the dimensions of EI are better predictors of physical or mental health.

As dependent variables, we assessed physical and mental health with a generic measure of health, and health behaviors with a measure of health-related behaviors.

## Materials and Methods

### Participants and Procedure

Participants were 855 undergraduate students, 188 male and 668 female, with a mean age of 34.27 years (SD = 9.61), and an age range between 18 and 64 years, volunteered to take part in this study.

There were 188 (22%) men in the sample, mean age 36.29 years (SD = 9.96), age range between 18 and 61 years; and 668 (78%) women, mean age 33.7 years (SD = 9.43), age range between 18 and 64 years. These people were recruited in the National Open University (UNED). The participants were not compensated for taking part in the study.

### Measures

#### Trait EI Measures

##### Trait Meta-Mood Scale (Salovey et al., 1995)

The TMMS was designed to assess the way people reflect on their moods, and thus, it was conceived as an index of PEI (Salovey et al., 2002). The scale has three factors that provide three subscale scores: Attention or Attention to Feelings, which evaluates the extent to which people attend to and value their emotions and moods; Clarity or Clarity of Feelings, relating to the ability to discriminate between emotions and moods, to feel clear rather than confused about one’s feelings; and Repair or Mood Repair, relating to the ability to regulate unpleasant moods or maintain pleasant modos and to using positive thinking to repair negative moods.

We used the well-validated Spanish shorter version of the TMMS (Fernández-Berrocal et al., 2004), which includes 24 items from the original version (eight for each subscale). The original 48 items were subjected to a principal components analysis with a varimax rotation. The analysis showed a three-factor solution with Attention, Clarity and Repair as dimensions, consistent with the findings of Salovey et al. (1995) for the English version. This Spanish version has shown aceptable internal consistency and satisfactory test–retest reliability. Further details on the scoring, reliability, and validity of the Spanish version of TMMS can be found in Fernández-Berrocal et al. (2004).

The final Spanish version consists of three subscales, as in the original version, each measuring different aspects of PEI: Attention (eight items corresponding to Items 7, 8, 13, 14, 35, 38, 41, and 46 of the English version), Clarity (eight items corresponding to Items 9, 12, 19, 26, 37, 42, 45, and 48 of the English version), and Repair (eight items corresponding to Items 2, 3, 6, 10, 16, 17, 40, and 43 of the English version; Fernández-Berrocal et al., 2004). In the final version of the TMMS, participants rate the extent to which they agreed with each item on a 5-point Likert-type scale ranging from 1 (Strongly disagree) to 5 (Strongly agree).

**Table 1** shows the results of internal consistency and the descriptive statistics of the three factors of this scale in the population of our study.

**Table 1:** Cronbach’s alphas, means, SDs of the variables examined.

	Total sample *N* = 855			Male *N* = 188		Female *N* = 667	
Scale (number of ítems)	Cronbach Alpha	*M*	SD	*M*	SD	*M*	SD
**Trait Meta-Mood Scale (TMMS)**
Attention (8)	0.89	27.32	6.29	25.45	6.20	27.85	6.21
Clarity (8)	0.90	28.27	6.10	29.00	6.50	28.07	5.97
Repair (8)	0.87	28.29	6.15	29.21	5.87	28.03	6.20
**Trait emotional intelligence questionnaire (TEIQue)**
Well-Being (3)	0.86	5.27	0.94	5.33	0.89	5.25	0.95
Emotionality (4)	0.73	5.28	0.70	5.10	0.74	5.33	0.69
Sociability (3)	0.77	4.76	0.75	4.89	0.80	4.72	0.73
Self-Control (3)	0.82	4.66	0.84	4.96	0.84	4.58	0.82
Global Trait EI	0.90	5.01	0.63	5.06	0.67	4.99	0.62
**Health survey SF-36 questionnaire (SF-36)**
Physical Functioning (10)	0.83	28.74	2.26	29.29	1.83	28.58	2.34
Role Physical (4)	0.92	17.84	3.15	18.43	2.69	17.67	3.25
Bodily Pain (2)	0.76	8.73	2.05	9.28	1.77	8.57	2.10
General Health (5)	0.81	19.98	3.52	20.63	3.16	19.79	3.60
Vitality (4)	0.83	14.20	3.07	15.20	2.72	13.92	3.10
Social Functioning (2)	0.83	8.64	1.75	9.03	1.48	8.53	1.81
Role Emotional (3)	0.91	13.03	2.55	13.32	2.38	12.94	2.59
Mental Health (5)	0.84	19.45	3.55	20.22	3.40	19.23	3.56
Physical Health Component (21)^a^	0.88	75.33	8.55	77.63	6.96	74.66	8.85
Mental Health Component (14)^b^	0.92	55.37	9.20	57.96	8.22	54.63	9.34
**Health behavior checklist (HBC)**
Wellness Maintenance and Enhancement Behaviors (10)	0.70	28.54	6.90	27.78	7.13	28.76	6.82
Accident Control Behaviors (6)	0.64	16.87	4.99	16.75	5.15	16.90	4.94
Preventive Health Behavior (16)^c^	0.77	45.41	10.22	44.53	10.53	45.66	10.13
Traffic Risk Taking (7)	0.65	17.09	4.68	18.14	5.20	16.80	4.48
Substance Risk Taking (3)	0.55	6.77	3.12	7.19	3.34	6.65	3.05
Risk Taking Behavior (10)^d^	0.62	23.85	6.02	25.33	6.68	23.43	5.76

^a^Physical Health Component is the sum of Physical Functioning, Role Physical, Bodily Pain, and General Health. ^b^Mental Health Component is the sum of Vitality, Social Functioning, Role Emotional and Mental Health. ^c^Preventive Health Behavior is the sum of Wellness Maintenance and Enhancement Behaviors and Accident Control Behaviors. ^d^Risk Taking Behavior is the sum of Traffic Risk Taking and Substance Risk Taking.

##### Trait emotional intelligence questionnaire (Petrides and Furnham, 2003a; Petrides, 2009a,b)

The TEIQue operationalizes the model of Petrides (Petrides and Furnham, 2001; Petrides et al., 2007c).

We used the most recent versión of this questionnaire, the TEIQue v 1.50 (Petrides, 2009a). This versión consists of 153 items (rated on a 7-point Likert scale) and 13 facets, organized in four factors: Well-being, Self-control, Emotionality, and Sociability. Two additional facets (Adaptability, Self-motivation) contribute directly to the Global Trait EI score.

The Well-Being factor refers to a generalized sense of wellbeing extending from past achievements to future expectations, accompanied by high self-esteem, and includes the facets of self-esteem, trait happiness, and trait optimism.

The Emotionality factor reflects the ability to identify and express feelings, and to use these faculties to maintain close relationships with significant others, and it includes the facets of emotion perception, emotion expression, trait empathy, and relationships.

The Sociability factor, regarding the capacity to assert oneself as well as to influence others’ emotions and decisions, includes the facets of social awareness, emotion management, and assertiveness.

The Self-Control factor, concerning the ability to regulate one’s impulses and emotions, as well as managing external pressures and stress, includes the facets of emotion regulation, stress management and impulsiveness.

In the 1.50 version of the TEIQue, participants rate their degreee of agreement with each ítem on a 7-point Likert-type scale ranging from 1 (Completely disagree) to 7 (Completely agree).

Regarding the validation of trait EI, Petrides and colleagues (Petrides and Furnham, 2001, 2003b; Petrides et al., 2007c) found an oblique trait EI factor both in Eysenckian and the Big Five factor space. Trait EI therefore provides evidence of its discriminant validity versus well-established personality dimensions.

The instrument has shown excellent psychometric properties in a series of studies (for detailed psychometric analyses of the TEIQue, see Mikolajczak et al., 2007; Freudenthaler et al., 2008; Jolic-Marjanovic and Altaras-Dimitrijevic, 2014).

**Table 1** presents the results of internal consistency and descriptive statistics of the four factors that make up this questionnaire in the population of our study.

#### Health Measures

##### Health survey SF-36 questionnaire (SF-36; Ware and Sherbourne, 1992)

This instrument was developed from the Medical Outcome Study (MOS; Ware and Sherbourne, 1992), and measures concepts that represent excellent basic human values for health. It is applicable to the general population as well as to clinical groups (McHorney et al., 1992, 1994). The SF-36 is a generic measure of health status as opposed to one that targets a specific age, disease, or treatment group (Ware and Gandek, 1998).

It is comprised of 36 items that report positive and negative states of physical health and emotional well-being. It identifies eight dimensions of health: Physical Functioning, Role Physical, Bodily Pain, General Health, Vitality, Social Functioning, Role Emotional, and Mental Health. Subsequently, a new dimension has been included, called Health Transition, which refers to the changes in the perception of the present state of health compared to how it was a year ago. Higher scores indicate a better state of health and/or a better quality of life in different areas (e.g., Physical Functioning, Mental Health, Vitality). Summary scores for a Physical Health Component (Physical Functioning, Role Physical, Bodily Pain, and General Health) and a Mental Health Component (Vitality, Social Functioning, Role Emotional, and Mental Health) can also be derived.

The reliability and validity of the SF-36 have been well documented by the developers of the instrument (Ware and Sherbourne, 1992; Ware et al., 1995).

We applied the versión that asks participants about all the health dimensions of the past 4 weeks, except for those of physical functioning and general health.

We used a Spanish version, which has shown good internal consistency, reliability, and validity in clinical samples (Alonso et al., 1995, 1998).

**Table 1** shows the results of internal consistency and the descriptive statistics of the eight scales of this questionnaire and the two general health components in the population of this study.

##### Health behavior checklist (Vickers et al., 1990)

This is a 40-item scale designed to assess health behaviors. Twenty-six of the items assess four factor-analytically derived health behaviors (see Vickers et al., 1990, for instrument content). The HBC measures four replicable factors: Wellness Maintenance and Enhancement Behaviors, consisting of 10 items (e.g., “I exercise to stay healthy”); Accident Control Behaviors, with six items (e.g., “I fix broken things around my house right away”); Traffic Risk Taking, with seven items (e.g., “I speed while driving”); and Substance Risk Taking, which consists of three the ítems (e.g., “I do not drink alcohol”). Participants indicate how well each item describes their typical behavior on a 5-point Likert scale ranging from 1 (Disagree strongly) to 5 (Agree strongly).

Health behaviors form two broader categories or dimensions: Preventive Health Behavior and Risk Taking Behavior. Preventive Health Behavior is the sum of the scores of Wellness Maintenance and Enhancement Behaviors and Accident Control Behaviors. Risk Taking Behavior is the sum of the scores of Traffic Risk Taking and Substance Risk Taking. In the categories of Traffic Risk Taking, Substance Risk Taking, and the broad category Risk Taking Behavior, a higher score indicates greater risk.

The procedures used to develop the HBC are described in Vickers et al. (1990), as well as the reliability and validity of the scale. There is also evidence of criterion-referenced validity in comparison with relevant measures (Vickers et al., 1990; Booth-Kewley and Vickers, 1994).

**Table 1** presents the results of internal consistency and the descriptive statistics of the four factors of this scale, and of the two broad categories from the population of this study.

#### Statistical Analysis

We used SPSS (version 20) for all data analyses. Correlations were reported as Pearson product moment correlations (two-tailed) for all continuous variables. To explore the predictive value of the EI dimensions as the independent variables, stepwise multiple regression analysis were performed, with the components of physical and mental health and the categories of health-related behaviors as the dependent variables.

## Results

### Internal Consistencies and Descriptive Statistics

Cronbach alphas, means, and SDs were calculated for each scale. **Table 1** present the results for the total sample of participants, and by gender. All the internal consistency values are within aceptable levels.

### Correlations Between the EI Dimensions and the Physical and Mental Health Components

To test the relationship between the EI dimensions and the health components, Pearson product moment correlations were computed between the dimensions of the EI measures (TMMS, TEIQue) and the physical and mental health components (SF-36). We also examined the relation between participants’ age and the health components.

As shown in **Table 2**, in general, almost all the EI dimensions had a significant and positive correlation with the two broad Physical and Mental Health Components, and also with the specific components, except for the Attention dimension of TMMS, which had significant but negative correlations with these components.

**Table 2:** Pearson correlations between the emotional intelligence (EI) dimensions and the Health Components.

Health survey SF-36 questionnaire (SF-36)										
	Physical Functioning	Role Physical	Bodily Pain	General Health	Vitality	Social Functioning	Role Emotional	Mental Health	Physical Health Component^a^	Mental Health Component^b^
**Trait Meta-Mood Scale (TMMS)**										
Attention	-0.023 (*p* = 0.510)	-0.130^∗∗∗^ (*p* = 0.000)	-0.111^∗∗∗^ (*p* = 0.001)	-0.161^∗∗∗^ (*p* = 0.000)	-0.213^∗∗∗^ (*p* = 0.000)	-0.277^∗∗∗^ (*p* = 0.000)	-0.318^∗∗∗^ (*p* = 0.000)	-0.311^∗∗∗^ (*p* = 0.000)	-0.153^∗∗∗^ (*p* = 0.000)	-0.331^∗∗∗^ (*p* = 0.000)
Clarity	0.062 (*p* = 0.069)	0.041 (*p* = 0.231)	0.075^∗^ (*p* = 0.029)	0.146^∗∗∗^ (*p* = 0.000)	0.206^∗∗∗^ (*p* = 0.000)	0.152^∗∗∗^ (*p* = 0.000)	0.086^∗^ (*p* = 0.012)	0.208^∗∗∗^ (*p* = 0.000)	0.104^∗∗^ (*p* = 0.003)	0.194^∗∗∗^ (*p* = 0.000)
Repair	0.086^∗^ (*p* = 0.012)	0.052 (*p* = 0.130)	0.082^∗^ (*p* = 0.017)	0.238^∗∗∗^ (*p* = 0.000)	0.314^∗∗∗^ (*p* = 0.000)	0.230^∗∗∗^ (*p* = 0.000)	0.226^∗∗∗^ (*p* = 0.000)	0.348^∗∗∗^ (*p* = 0.000)	0.160^∗∗∗^ (*p* = 0.000)	0.344^∗∗∗^ (*p* = 0.000)
**Trait emotional intelligence questionnaire (TEIQue)**										
Well-Being	0.179^∗∗∗^ (*p* = 0.000)	0.182^∗∗∗^ (*p* = 0.000)	0.189^∗∗∗^ (*p* = 0.000)	0.438^∗∗∗^ (*p* = 0.000)	0.544^∗∗∗^ (*p* = 0.000)	0.418^∗∗∗^ (*p* = 0.000)	0.425^∗∗∗^ (*p* = 0.000)	0.647^∗∗∗^ (*p* = 0.000)	0.345^∗∗∗^ (*p* = 0.000)	0.623^∗∗∗^ (*p* = 0.000)
Emotionality	0.035 (*p* = 0.314)	0.088^∗∗^ (*p* = 0.010)	0.082^∗^ (*p* = 0.017)	0.224^∗∗∗^ (*p* = 0.000)	0.310^∗∗∗^ (*p* = 0.000)	0.255^∗∗∗^ (*p* = 0.000)	0.232^∗∗∗^ (*p* = 0.000)	0.356^∗∗∗^ (*p* = 0.000)	0.153^∗∗∗^ (*p* = 0.000)	0.344^∗∗∗^ (*p* = 0.000)
Sociability	0.072^∗^ (*p* = 0.035)	0.054 (*p* = 0.114)	0.069^∗^ (*p* = 0.046)	0.191^∗∗∗^ (*p* = 0.000)	0.303^∗∗∗^ (*p* = 0.000)	0.205^∗∗∗^ (*p* = 0.000)	0.144^∗∗∗^ (*p* = 0.000)	0.306^∗∗∗^ (*p* = 0.000)	0.127^∗∗∗^ (*p* = 0.000)	0.294^∗∗∗^ (*p* = 0.000)
Self-Control	0.128^∗∗∗^ (*p* = 0.000)	0.198^∗∗∗^ (*p* = 0.000)	0.159^∗∗∗^ (*p* = 0.000)	0.332^∗∗∗^ (*p* = 0.000)	0.427^∗∗∗^ (*p* = 0.000)	0.380^∗∗∗^ (*p* = 0.000)	0.411^∗∗∗^ (*p* = 0.000)	0.585^∗∗∗^ (*p* = 0.000)	0.288^∗∗∗^ (*p* = 0.000)	0.549^∗∗∗^ (*p* = 0.000)
**Age**	-0.145*** (*p* = 0.000)	0.008 (*p* = 0.823)	-0.001 (*p* = 0.979)	0.090** (*p* = 0.010)	0.168*** (*p* = 0.000)	0.112*** (*p* = 0.001)	0.223*** (*p* = 0.000)	0.173*** (*p* = 0.000)	0.002 (*p* = 0.952)	0.204*** (*p* = 0.000)

**Health behavior checklist (HBC)**										
		**Wellness Maintenance and Enhancement Behaviors**		**Accident Control Behaviors**		**Preventive Health Behavior^c^**		**Traffic Risk Taking**	**Substance Risk Taking**	**Risk Taking Behavior^d^**

**Trait Meta-Mood Scale (TMMS)**										
Attention		0.013 (*p* = 0.701)		-0.056 (*p* = 0.100)		-0.019 (*p* = 0.589)		0.066 (*p* = 0.056)	0.045 (*p* = 0.195)	0.074^∗^ (*p* = 0.031)	
Clarity		0.124^∗∗∗^ (*p* = 0.000)		0.206^∗∗∗^ (*p* = 0.000)		0.184^∗∗∗^ (*p* = 0.000)		-0.051 (*p* = 0.137)	-0.068^∗^ (*p* = 0.047)	-0.074^∗^ (*p* = 0.030)
Repair		0.156^∗∗∗^ (*p* = 0.000)		0.176^∗∗∗^ (*p* = 0.000)		0.191^∗∗∗^ (*p* = 0.000)		-0.057 (*p* = 0.100)	-0.015 (*p* = 0.668)	-0.051 (*p* = 0.137)
**Trait emotional intelligence questionnaire (TEIQue)**										
Well-Being		0.210^∗∗∗^ (*p* = 0.000)		0.162^∗∗∗^ (*p* = 0.000)		0.221^∗∗∗^ (*p* = 0.000)		-0.032 (*p* = 0.348)	-0.060 (*p* = 0.079)	-0.056 (*p* = 0.104)
Emotionality		0.183^∗∗∗^ (*p* = 0.000)		0.224^∗∗∗^ (*p* = 0.000)		0.233^∗∗∗^ (*p* = 0.000)		-0.145^∗∗∗^ (*p* = 0.000)	-0.096^∗∗^ (*p* = 0.005)	-0.162^∗∗∗^ (*p* = 0.000)
Sociability		0.125^∗∗∗^ (*p* = 0.000)		0.158^∗∗∗^ (*p* = 0.000)		0.161^∗∗∗^ (*p* = 0.000)		0.045 (*p* = 0.186)	0.003 (*p* = 0.937)	0.037 (*p* = 0.286)
Self-Control		0.101^∗∗^ (*p* = 0.003)		0.190^∗∗∗^ (*p* = 0.000)		0.161^∗∗∗^ (*p* = 0.000)		-0.143^∗∗∗^ (*p* = 0.000)	-0.142^∗∗∗^ (*p* = 0.000)	-0.184^∗∗∗^ (*p* = 0.000)
**Age**		0.152*** (*p* = 0.000)		0.175*** (*p* = 0.000)		0.188*** (*p* = 0.000)		-0.192*** (*p* = 0.000)	-0.107** (*p* = 0.002)	-0.207*** (*p* = 0.000)

Total sample *N* = 855, ***p ≤ 0.001 (two-tailed); **p ≤ 0.01 (two-tailed); *p ≤ 0.05 (two-tailed). ^a^Physical Health Component is the sum of Physical Functioning, Role Physical, Bodily Pain and General Health. ^b^Mental Health Component is the sum of Vitality, Social Functioning, Role Emotional and Mental Health. ^c^Preventive Health Behavior is the sum of Wellness Maintenance and Enhancement Behaviors and Accident Control Behaviors. ^d^Risk Taking Behavior is the sum of Traffic Risk Taking and Substance Risk Taking.

The only dimensions with no significant relations were: Attention, Clarity, and Emotionality did not correlate with Physical Functioning; Clarity, Repair, and Sociability did not correlate with Role Physical.

Age presented significant and positive relationship with General Health, Vitality, Social Functioning, Role Emotional, Mental Health, and with the broad Mental Health Component; but its relation with Physical Functioning was negative and it did not correlate with Role Physical, Bodily Pain, or with the broad Physical Health Component.

### Correlations Between the EI Dimensions and the Categories of Health-Related Behaviors

To test the relationship between the EI dimensions and health-related behaviors, Pearson product moment correlations were computed between the EI dimensions (TMMS, TEIQue) and these categories (HBC). We also examined the relation between participants’ age and the health-related behaviors.

As shown in **Table 2**, almost all the EI dimensions had significant and positive relations with the specific categories of Wellness Maintenance and Enhancement Behaviors, and Accident Control Behaviors, and with the broad category Preventive Health Behavior. The EI dimensions also had significant and negative correlations with the specific categories of Traffic Risk Taking, and Substance Risk Taking, and with the broad category Risk Taking Behavior, except for the Attention dimension, which had a positive relation with the broad category of Risk Taking Behavior. Attention had no significant correlations with any of the specific health-related behaviors.

Clarity did not correlate with Traffic Risk Taking. Repair, Well-Being, and Sociability had no significant relation with Traffic Risk Taking, Substance Risk Taking, or with the broad category Risk Taking Behavior.

Age had significant relationships with all the health-related behaviors, which were positive in the case of Wellness Maintenance and Enhancement Behaviors, Accident Control Behaviors, and with the broad category Preventive Health Behavior, and negative in the case of Traffic Risk Taking, Substance Risk Taking, and the broad category Risk Taking Behavior.

### Stepwise Multiple Regression with EI Dimensions as Predictor Variables, and Criterial Variables Each One of the Physical and Mental Health Components

Prior to the stepwise multiple regression analysis, the relationships between independent variables (TMMS, TEIQue) and the dependent variables (SF-36) were examined. Independent variables significantly associated with the physical and mental health components were considered candidate predictors and were entered into the stepwise multiple regression analysis.

In order to avoid the collinearity problem with the TEIQue factors, and also because our focus was on the study of the EI dimensions and not on general EI, we did not enter the general EI measure from this questionnaire in any analysis.

We followed the same procedure as with the EI dimensions with the independent variable age, considering it an independent variable in the situations in which it had a significant relation with some health component.

In all the analyses, gender was entered as an independent variable to determine whether it predicted health. The assigned code for the analyses was 1 = men and 2 = women.

In this type of regression analysis, the sign of the partial regression coefficient (ß) of a variable should not be the same as the sign of the simple correlation coefficient between that variable and the dependent variable because of the adjustments made to obtain the best possible equation.

The results are presented in **Table 3**. In general, the EI dimensions predicted mental health more strongly than physical health. Starting with the two broad health components, the EI dimensions explained the Mental Health Component better than they explained the Physical Health Component.

**Table 3:** Stepwise multiple regression analysis.

Model	*R*	*R*^2^	*R*^2 ^adjusted	*R*^2 ^change	*F*(df)	β	β standarized	*t*
**Dependent variables: components of the health survey SF-36 questionnaire (SF-36)**								
**Independent variables: dimensions of the Trait Meta-Mood Scale (TMMS) and Trait Emotional Intelligence Questionnaire (TEIQue)**								

**Dependent variable: Physical Functioning**								
Model 1: Well-Being	0.178	0.032	0.031	0.032	27.317 (1,853)***	0.436	0.178	5.232***
Model 2: Well-Being						0.518	0.212	6.218***
Age	0.254	0.064	0.062	0.033	28.814 (2,852)***	-0.044	-0.184	-5.416***
Model 3: Well-Being						0.512	0.209	6.206***
Age						-0.047	-0.199	-5.889***
Gender	0.291	0.085	0.082	0.020	25.820 (3,851)***	-0.787	-0.144	-4.315***

**Dependent variable: Role Physical**								
Model 1: Self-Control	0.200	0.040	0.039	0.040	34.901 (1,853)***	0.748	0.200	5.908***
Model 2: Self-Control						0.534	0.143	3.591***
Well-Being	0.220	0.049	0.046	0.008	21.263 (2,852)***	0.367	0.108	2.713**
Model 3: Self-Control						0.446	0.120	2.913**
Well-Being						-0.041	-0.081	-2.286*
Attention	0.233	0.054	0.051	0.006	15.990 (3,851)***	0.365	0.107	2.705**

**Dependent variable: Bodily Pain**								
Model 1: Well-Being	0.180	0.033	0.031	0.033	28.095 (1,853)***	0.400	0.180	5.300***
Model 2: Well-Being						0.388	0.175	5.178***
Gender	0.224	0.050	0.048	0.018	22.109 (2,852)***	-0.662	-0.134	-3.954***

**Dependent variable: General Health**								
Model 1: Well-Being	0.424	0.180	0.179	0.180	179.839 (1,853)***	1.595	0.424	13.410***
Model 2: Well-Being						1.322	0.352	9.470***
Self-Control	0.439	0.193	0.191	0.013	97.960 (2,852)***	0.566	0.136	3.656***
Model 3: Well-Being						1.488	0.396	9.632***
Self-Control						0.609	0.146	3.925***
Sociability	0.446	0.199	0.196	0.006	67.749 (3,851)***	-0.434	-0.092	-2.471*
Model 4: Well-Being						1.519	0.404	9.809***
Self-Control						5.48	0.132	3.475***
Sociability						-0.459	-0.098	-2.612**
Gender	0.451	0.203	0.199	0.004	52.101 (4,850)***	-0.557	-0.066	-2.081*

**Dependent variable: Vitality**								
Model 1: Well-Being	0.540	0.291	0.290	0.291	342.622 (1,853)***	1.788	0.540	18.510***
Model 2: Well-Being						1.450	0.437	12.925***
Self-Control	0.563	0.317	0.316	0.026	193.728 (2,852)***	0.696	0.192	5.663***
Model 3: Well-Being						1.488	0.449	13.360***
Self-Control						0.587	0.162	4.718***
Gender	0.576	0.332	0.330	0.014	137.811 (3,851)***	-0.910	-0.123	-4.248***
Model 4: Well-Being						1.485	0.448	13.374***
Self-Control						0.509	0.140	3.997***
Gender						-0.851	-0.115	-3.967***
Attention	0.581	0.338	0.334	0.006	105.877 (4,850)***	-0.039	-0.079	-2.658**

**Dependent variable: Social Functioning**								
Model 1: Well-Being	0.406	0.165	0.164	0.165	164.231 (1,853)***	0.764	0.406	12.815***
Model 2: Well-Being						0.700	0.372	11.886***
Attention	0.456	0.208	0.206	0.043	108.715 (2,852)***	-0.058	-0.209	-6.677***
Model 3: Well-Being						0.540	0.287	7.978***
Attention						-0.048	-0.172	-5.379***
Self-Control	0.477	0.228	0.225	0.020	81.509 (3,851)***	0.358	0.173	4.655***

**Dependent variable: Role Emotional**								
Model 1: Well-Being	0.421	0.177	0.176	0.177	180.389 (1,853)***	1.152	0.421	13.431***
Model 2: Well-Being						1.040	0.380	12.439***
Attention	0.489	0.239	0.237	0.062	131.350 (2,852)***	-0.102	-0.252	-8.239***
Model 3: Well-Being						0.762	0.279	7.975***
Attention						-0.084	-0.208	-6.698***
Self-Control	0.517	0.268	0.265	0.028	101.608 (3,851)***	0.617	0.205	5.682***
Model 4: Well-Being						0.920	0.336	8.699***
Attention						-0.078	-0.194	-6.225***
Self-Control						0.670	0.223	6.145***
Sociability	0.527	0.278	0.274	0.010	80.044 (4,850)***	-0.411	-0.120	-3.393***
Model 5: Well-Being						0.895	0.327	8.486***
Attention						-0.073	-0.181	-5.815***
Self-Control						0.632	0.210	5.804***
Sociability						-0.412	-0.121	-3.427***
Age	0.536	0.287	0.283	0.009	66.962 (5,849)***	0.026	0.100	3.294***

**Dependent variable: Mental Health**								
Model 1: Well-Being	0.640	0.410	0.409	0.410	578.526 (1,853)***	2.432	0.640	24.053***
Model 2: Well-Being						1.745	0.459	15.800***
Self-Control	0.703	0.494	0.492	0.084	406.000 (2,852)***	1.429	0.342	11.758***
Model 3: Well-Being						1.741	0.458	16.039***
Self-Control						1.256	0.300	10.172***
Attention	0.715	0.512	0.510	0.018	290.434 (3,851)***	-0.079	-0.140	-5.525***
Model 4: Well-Being						1.892	0.498	15.739***
Self-Control						1.307	0.313	10.523***
Attention						-0.073	-0.130	-5.112***
Sociability	0.719	0.516	0.514	0.005	221.801 (4,850)***	-0.396	-0.084	-2.877**

**Dependent variable: Physical Health Component^a^**								
Model 1: Well-Being	0.337	0.114	0.113	0.114	104.464 (1,853)***	3.097	0.337	10.221***
Model 2: Well-Being						3.049	0.332	10.143***
Gender	0.361	0.130	0.128	0.016	60.770 (2,852)***	-2.618	-0.128	-3.905***
Model 3: Well-Being						2.473	0.269	6.976***
Gender						-2.219	-0.108	-3.263***
Self-Control	0.374	0.140	0.137	0.010	43.968 (3,851)***	1.206	0.119	3.024**
Model 4: Well-Being						3.005	0.327	7.634***
Gender						-2.348	-.115	-3.464***
Self-Control						1.322	0.130	3.317***
Sociability	0.387	0.149	0.145	0.010	35.628 (4,850)***	-1.360	-0.118	-3.044**
Model 5: Well-Being						3.088	0.336	7.848***
Gender						-2.487	-0.121	-3.670***
Self-Control						1.443	0.142	3.608***
Sociability						-1.374	-0.120	-3.086**
Age	0.396	0.156	0.151	0.007	30.047 (5,849)***	-0.077	-0.086	-2.592**

**Dependent variable: Mental Health Component^b^**								
Model 1: Well-Being	0.617	0.381	0.380	0.381	506.709 (1,853)***	6.084	0.617	22.510***
Model 2: Well-Being						4.472	0.454	14.863***
Self-Control	0.670	0.449	0.448	0.068	335.442 (2,852)***	3.341	0.308	10.099***
Model 3: Well-Being						4.459	0.452	15.193***
Self-Control						2.781	0.257	8.334***
Attention	0.690	0.477	0.475	0.027	249.324 (3,851)***	-0.253	-0.173	-6.549***
Model 4: Well-Being						4.790	0.486	14.742***
Self-Control						2.901	0.268	8.615***
Attention						-0.241	-0.165	-6.177***
Sociability	0.693	0.480	0.478	0.003	189.387 (4,850)***	-0.871	-0.071	-2.343*
Model 5: Well-Being						4.871	0.494	14.942***
Self-Control						2.767	0.255	8.120***
Attention						-0.231	-0.158	-5.901***
Sociability						-0.939	-0.076	-2.524*
Gender	0.695	0.484	0.480	0.003	153.342 (5,849)***	-1.307	-0.059	-2.290*

**Dependent variables: categories of the health behavior checklist (HBC)**								
**Independent variables: dimensions of the Trait Meta-Mood Scale (TMMS) and Trait Emotional Intelligence Questionnaire (TEIQue)**								

**Dependent variable: Wellness Maintenance and Enhancement Behaviors**								

Model 1: Well-Being	0.206	0.042	0.041	0.042	37.124 (1,853)***	1.535	0.206	6.093***
Model 2: Well-Being						1.372	0.184	5.392***
Age	0.238	0.057	0.054	0.014	25.071 (2,852)***	0.087	0.121	3.537***
Model 3: Well-Being						1.382	0.185	5.444***
Age						0.093	0.129	3.767***
Gender	0.251	0.063	0.059	0.006	18.660 (3,851)***	1.325	0.079	2.359*
Model 4: Well-Being						1.233	0.166	4.678***
Age						0.091	0.127	3.709***
Gender						1.381	0.083	2.458*
Clarity	0.260	0.067	0.063	0.005	15.078 (4,850)***	0.081	0.071	2.031*

**Dependent variable: Accident Control Behaviors**								
Model 1: Emotionality	0.221	0.049	0.048	0.049	42.945 (1,853)***	1.566	0.221	6.553***
Model 2: Emotionality						1.418	0.200	5.936***
Age	0.264	0.070	0.068	0.021	31.362 (2,852)***	0.076	0.146	4.343***
Model 3: Emotionality						0.975	0.137	3.498***
Age						0.075	0.145	4.323***
Clarity	0.283	0.080	0.077	0.010	24.224 (3,851)***	0.098	0.119	3.054**
Model 4: Emotionality						0.771	0.109	2.615**
Age						0.069	0.133	3.917***
Clarity						0.091	0.111	2.827*
Self-Control	0.291	0.085	0.080	0.005	19.327 (4,850)***	0.465	0.079	2.084*

**Dependent variable: Preventive Health Behavior^c^**								
Model 1: Emotionality	0.228	0.052	0.051	0.052	46.071 (1,853)***	3.327	0.228	6.788***
Model 2: Emotionality						2.996	0.206	6.127***
Age	0.278	0.077	0.075	0.025	34.948 (2,852)***	0.170	0.160	4.757***
Model 3: Emotionality						2.427	0.167	4.612***
Age						0.159	0.149	4.441***
Repair	0.293	0.086	0.083	0.009	26.205 (3,851)***	0.174	0.103	2.850**

**Dependent variable: Traffic Risk Taking**								
Model 1: Age	0.190	0.036	0.035	0.036	31.597 (1,853)***	-0.093	-0.190	-5.621***
Model 2: Age						-0.100	-0.206	-6.101***
Gender	0.237	0.056	0.054	0.020	24.896 (2,852)***	-1.599	-0.141	-4.192***
Model 3: Age						-0.088	-0.180	-5.262***
Gender						-1.856	-0.164	-4.835***
Self-Control	0.270	0.073	0.070	0.017	22.028 (3,851)***	-0.754	-0.136	-3.929***

**Dependent variable: Substance Risk Taking**								
Model 1: Self-Control	0.146	0.021	0.020	0.021	18.141 (1,853)***	-.540	-0.146	-4.259***
Model 2: Self-Control						-0.612	-0.165	-4.759***
Gender	0.177	0.031	0.029	0.010	13.442 (2,852)***	-0.767	-0.102	-2.929**
Model 3: Self-Control						-0.546	-0.147	-4.180***
Gender						-0.812	-0.108	-3.103**
Age	0.196	0.038	0.035	0.007	11.133 (3,851)***	-0.029	-0.088	-2.518*

**Dependent variable: Risk Taking Behavior^d^**								
Model 1: Age	0.208	0.043	0.042	0.043	37.775 (1,853)***	-0.130	-0.208	-6.146***
Model 2: Age						-0.140	-0.224	-6.661***
Gender	0.257	0.066	0.064	0.023	29.539 (2,852)***	-2.207	-0.152	-4.519***
Model 3: Age						-0.118	-0.189	-5.585***
Gender						-2.654	-0.183	-5.438***
Self-Control	0.310	0.096	0.093	0.030	29.659 (3,851)***	-1.290	-0.181	-5.291***

Total sampleN = 855, ***p ≤ 0.001; **p ≤ 0.01; *p ≤ 0.05. ^a^Physical Health Component is the sum of Physical Functioning, Role Physical, Bodily Pain and General Health. ^b^Mental Health Component is the sum of Vitality, Social Functioning, Role Emotional and Mental Health. ^c^Preventive Health Behavior is the sum of Wellness Maintenance and Enhancement Behaviors and Accident Control Behaviors. ^d^Risk Taking Behavior is the sum of Traffic Risk Taking and Substance Risk Taking.

Regarding the broad health component, Physical Health Component, the prediction model contained five predictors and was reached in five steps, *F*(5,849) = 30.047, *p* < 0.001, accounting for 15.6% of the variance of the Physical Health Component (*R*^2^ = 0.156). The significant predictors of this model were Well-Being (*R*^2^ = 0.114), Gender (*R*^2^ = 0.016), Sefl-Control (*R*^2^ = 0.01), Sociability (*R*^2^ = 0.01), and Age (*R*^2^ = 0.007), with the men obtaining higher scores than the women in this component (see the mean score of men and women in **Table 1**).

Regarding the Mental Health Component, the model contained five predictors and include five steps, *F*(5,849) = 153.342, *p* < 0.001, accounting for 48.4% of the variance of this component (*R*^2^ = 0.484). The significant predictors of this model were Well-Being (*R*^2^ = 0.381), Self-Control (*R*^2^ = 0.068), Attention (*R*^2^ = 0.027; the relation is negative, see **Table 2**), Sociability (*R*^2^ = 0.003), and Gender (*R*^2^ = 0.003). The men presented higher scores than the women in the Mental Health Component (see the mean score of men and women in **Table 1**). The results are similar to those of the Physical Health Component, except that in the Mental Health Component Age was not a predictor, but Attention was.

Regarding the specific health components, Mental Health, the model *F*(4,850) = 221.801, *p* < 0.001, accounted for approximately 52% of the variance of the Mental Health (*R*^2^ = 0.516). Significant predictors were Well-Being (*R*^2^ = 0.410), Self-Control (*R*^2^ = 0.084), Attention (*R*^2^ = 0.018; the relation was negative, see **Table 2**), and Sociability (*R*^2^ = 0.005).

The EI dimensions also predicted a considerable percentage of the variance of Vitality 33.8% (*R*^2^ = 0.338), Role Emotional 28.7% (*R*^2^ = 0.287), Social Functioning 22.8% (*R*^2^ = 0.228), and General Health 20.3% (*R*^2^ = 0.203).

As seen in **Table 3**, the EI dimensions that do not emerge as predictors of health components are Clarity, Repair, and Emotionality. The EI dimension that most strongly predicts health behaviors is Well-Being, followed by Attention and Self-Control, but predicting a much lower percentage.

### Stepwise Multiple Regression with EI Dimensions as Predictor Variables, and Criterial Variables Each One of the Categories of Health-Related Behaviors

The same procedure as in the former stepwise multiple regression analyses was followed to select the possible predictors. In these analyses, the dependent variables were each one of the categories of the HBC scale. The same procedure was followed to enter age into the analyses. Gender was entered in all the analyses; the assigned code was 1 = men and 2 = women.

As in the analyses with the health components, we did not enter general the measure of EI from the TEIQue questionnaire in the analyses to avoid the colinearity problem with the TEIQue factors, and also because our focus was on the study of the EI dimensions and not on general EI.

The results can be seen in **Table 3**. EI dimensions predict almost equally the two broad categories of health-related behaviors, Preventive Health Behavior and Risk Taking Behavior.

In the broad category of Risk Taking Behavior, the prediction model contained three predictors and was reached in three steps, *F*(3,851) = 29.659, *p* < 0.001, accounted for 9.6% of the variance (*R*^2^ = 0.096). Significant predictors of this model were Age (*R*^2^ = 0.043; the relation was negative, see **Table 2**), Gender (*R*^2^ = 0.023), with men assuming more risk behaviors related to traffic safety, and in consumption of substances such as alcohol and tobacco (see mean scores of men and women in **Table 1**). The only EI predictor was Self-Control (*R*^2^ = 0.030; the relation was negative, see **Table 2**).

In the other broad category, Preventive Health Behavior, the prediction model also contained three predictors, *F*(3,851) = 26.205, *p* < 0.001, accounting for 8.6% of the variance (*R*^2^ = 0.086). Significant predictors of this model were Emotionality (*R*^2^ = 0.052), Age (*R*^2^ = 0.025), and Repair (*R*^2^ = 0.009).

The EI dimensions of Attention and Sociability did not emerge as predictors of health-related behaviors. The EI dimensions of Emotionality and Well-Being, followed by Self-Control, were the ones that best predicted this type of behaviors.

## Conclusion

The current research investigated the EI dimensions that can explain various general, physical, and mental health components, and various categories of health-related behaviors.

Most of the studies carried out to verify the relation between EI and health have observed a relation with general EI. Nevertheless, some studies have used EI measures that provide scores in specific dimensions that predict health (Extremera and Fernández-Berrocal, 2002; Salovey et al., 2002; Tsaousis and Nikolaou, 2005; Johnson et al., 2009). In our study, we extended the number of EI dimensions by using two measures that assess different dimensions, and the results obtained provide sets of EI dimensions that predict health.

One of our goals was to obtain outcomes with the greatest possible number of EI dimensions in order to delimit those that best predict health and health-related behaviors. Hence, in our investigation, we extended the assessment of EI, applying the two most used measures, the TMMS and the TEIQue (Martins et al., 2010), which appraise different EI dimensions. Similarly, to expand the study to the greatest possible number of health components, we applied a general health questionnaire, the SF-36, which provides two general measures of physical and mental health, and various more specific health components.

In addition to this measure of general health, due to the scarcity of studies carried out to establish an association between EI and health-related behaviors, and between EI and addiction-related behaviors (Kun and Demetrovics, 2010; Martins et al., 2010), we also wished to appraise this relation in our study, applying the HBC, a scale specifically designed to assess healthy behaviors, which provides two broad categories, Preventive Health Behavior and Risk Taking Behavior, and four more specific factors.

From the regression analyses, it can be concluded that the EI dimensions analyzed are better predictors of mental health (48.4%) than of physical health (15.6%). The data obtained provide significant results to be able to delimit the specific EI dimensions most closely related to health.

Regarding the broad Mental Health Component, where a higher score predicts better mental health, the group of EI dimensions that explain it positively are Well-Being, Self-Control and Sociability, and negatively in the case of Attention.

The EI dimensions that positively explain the Physical Health Component, where a higher score predicts better physical health, are Well-Being, Self-Control and Sociability of the TEIQue.

With regard to TMMS, Extremera and Fernández-Berrocal (2002) also applied regression analysis to obtain the dimensions that best predict the health components, but we cannot compare our results with theirs because, in our study, we entered more EI dimensions as predictor variables. Nevertheless, some of the results are common: in both studies, the dimension of Attention coincides as a negative predictor of Role Physical, Vitality, Social Functioning, Role Emotional, and Mental Health.

Regarding the health-related behaviors, the results of the regression analyses, show that the EI dimensions predict a lower percentage of health-related behaviors than of health components. In the broad category, Risk Taking Behavior, the only EI dimension that predicts assuming health risks is Self-Control (TEIQue), a high score in this EI dimension predicts taking fewer risks. In the category of Preventive Health Behavior, two EI dimensions emerge that predict preventive behaviors, Emotionality and Repair.

As with the relation between EI and health, a single measure of EI is usually employed to examine the relation between EI and health-related behaviors, and only one or two unhealthy behaviors, such as alcohol and tobacco consumption, are tested (Austin et al., 2005; Tsaousis and Nikolaou, 2005; Limonero et al., 2006; Hill and Maggi, 2011). In this work, in addition to the results for the risk behaviors of alcohol and tobacco consumption, we also obtained results concerning traffic risk taking and preventive health-related behaviors.

Lastly, we note that gender emerges as a predictor of health, with the men obtaining higher values than the women both in the Mental Health and the Physical Health Component. Men also seem to display more health risk behaviors.

Age predicts fewer health risk behaviors, and it is a protector element, older people carry out more preventive behaviors for health.

These results can help future research to continue delimiting the specific EI dimensions that contribute to better health and to promoting the mechanisms through which emotional management can influence physical and mental health.

Regarding the data obtained from the correlations between the EI dimensions and the physical and mental health components, the results show that almost all the EI dimensions assessed are positively related to the Physical and Mental Health Components, except for Attention, which has a negative relation.

When examining the same relation between EI (TMMS) and health (SF-36), Extremera and Fernández-Berrocal (2002) only found a positive relation of the dimension of Attention with Role Emotional, but no relation was found with the remaining health components. Regarding the Clarity dimension, our results are similar to those obtained by these authors, although in our study, positive and significant relations were established with a larger number of health components. Lastly, these authors found a significant but negative relation between the Repair and Bodily Pain dimensions, and in our study, the relation between these dimensions was positive; that is, EI dimension of Repair is related to having fewer symptoms of bodily pain. This EI dimension has significant and positive relations with other health components, and our results are fairly similar to those obtained by these authors.

Salovey et al. (2002), found no relation between the Attention dimension and physical symptoms, but they did find a relation between the dimensions of Clarity and Repair with physical symptoms, both these EI dimensions were associated with lower levels of symptom reporting, social anxiety, and depression.

Freudenthaler et al. (2008), applying the TEIQue questionnaire, found a negative relation between somatic complaints, referring to various physical symptoms and bodily sensations scale, and the dimensions of Well-Being, Self-Control, and Sociability. Also with the TEIQue questionnaire, Mikolajczak et al. (2006) found a positive relation between physical and mental health and the four EI dimensions, Well-being, Self-Control, Emotionality, and Sociability. The results of these studies are similar to those obtained in this work, in which we verified that the dimensions of the TEIQue questionnaire present significant and positive relationships with the physical and mental health components.

In the results of this work, age had positive relations with almost all the components of physical and mental health, because these components—except for Physical Functioning and General Health—reflect information referring to the past 4 weeks before completing the questionnaire. Physical Functioning, which had a negative relation with age, appraises limitations to perform all kinds of physical activity, such as swimming, dressing, walking, squatting, going upstairs, lifting weights, and moderate and intense efforts. All these activities may be affected by age.

Regarding health-related behaviors, not all the EI dimensions are related to these behaviors. Attending to the two broad categories, all the EI dimensions except for Attention have a positive relation with Preventive Health Behavior, such that higher values in these dimensions imply preventive health behaviors. In the other broad category, Risk Taking Behavior, where a high score implies performing health risk behaviors, such as traffic risk taking, alcohol consumption, and smoking, not all the EI dimensions have a relation. Specifically, those that do not are Repair, Well-Being, and Sociability. The Attention dimension is positively related to this category, such that a high score in Attention is related to health risk behaviors, although the relation is not very high. The negatively related EI dimensions, in which a high score implies assuming fewer health risks, are Clarity, Emotionality, and Self-Control.

Tsaousis and Nikolaou (2005), examined the relation between EI and health behaviors, finding a negative relation between total EI and smoking and drinking, and a positive relation with exercising.

Limonero et al. (2006), applied the TMMS to determine the relation between EI and tobacco and cannabis use among university students, finding that the students who consumed tobacco or cannabis and who presented lower levels of the Repair dimension had started consuming tobacco or cannabis at an earlier age. The dimension of Clarity appears to be related to the occasional consumption of cannabis, such that the students obtaining high scores consumed less, whereas the dimension of Attention was not involved in the consumption of these substances. In our study, the dimension of Repair did not have a significant relation with health risk behaviors. Clarity presented a negative relation, and Attention was related to tobacco consumption in addition to other health risk behaviors.

Several limitations of the present study must be mentioned. This study was conducted with self-report measures, so it is likely that social desirability may have influenced the responses. Due to the cross-sectional design of our study, the assumption of causality should be considered with caution, and a follow-up longitudinal study would be valuable to address this limitation, so that future research using prospective designs is needed to confirm our findings.

Despite these limitations, this study provides preliminary evidence of some of the EI dimensions that could explain each one of the general components of physical and mental health, as well as some representative categories of health-related behaviors.

## Conflict of Interest Statement

The authors declare that the research was conducted in the absence of any commercial or financial relationships that could be construed as a potential conflict of interest.

## References

[B1] Alonso J. Prieto L. Antó J. M. (1995). La versión española del SF-36 health survey (Cuestionario de Salud SF-36): un instrumento para la medida de los resultados clínicos. *Med. Clín.* 104 771–776. 7783470

[B2] Alonso J. Prieto L. Ferrer M. Vilagut G. Broquetas J. M. Roca J. (1998). Testing the measurement properties of the Spanish version of the SF-36 health survey among male patients with chronic obstructive pulmonary disease. Quality of life in COPD study group. *J. Clin. Epidemiol.* 51 1087–1094 10.1016/S0895-4356(98)00100-0 9817126

[B3] Andrei F. Petrides K. V. (2013). Trait emotional intelligence and somatic complaints with reference to positive and negative mood. *Psihologija* 46 5–15 10.2298/PSI1301005A

[B4] Austin E. J. Saklofske D. H. Egan V. (2005). Personality, well-being and health correlates of trait emotional intelligence. *Pers. Individ. Diff.* 38 547–558 10.1016/j.paid.2004.05.009

[B5] Booth-Kewley S. Vickers R. R. (1994). Associations between major domains of personality and health behavior. *J. Pers.* 62 281–298 10.1111/j.1467-6494.1994.tb00298.x 7965560

[B6] Costa S. Petrides K. V. Tillmann T. (2014). Trait emotional intelligence and inflammatory diseases. *Psychol. Health Med.* 19 180–189 10.1080/13548506.2013.802356 23725416

[B7] Extremera N. Fernández-Berrocal P. (2002). Relation of perceived emotional intelligence and health-related quality of life of middle-aged women. *Psychol. Rep.* 9 47–59 10.2466/pr0.2002.91.1.47 12353803

[B8] Extremera N. Fernández-Berrocal P. (2005). Perceived emotional intelligence and life satisfaction: predictive and Incremental validity using the trait meta-mood scale. *Pers. Individ. Dif.* 39 937–948 10.1016/j.paid.2005.03.012

[B9] Fernández-Berrocal P. Extremera N. Ramos N. (2004). Validity and reliability of the Spanish modified version of the Trait Meta-Mood Scale. *Psychol. Rep.* 94 751–755 10.2466/pr0.94.3.751-755 15217021

[B10] Freudenthaler H. H. Neubauer A. C. Gabler P. Scherl W. G. Rindermann H. (2008). Testing and validating the trait emotional intelligence questionnaire (TEIQue) in a German-speaking simple. *Pers. Individ. Dif.* 45 673–678 10.1016/j.paid.2008.07.014

[B11] Goldman S. L. Kramer D. T. Salovey P. (1996). Beliefs about mood moderate the relationship of stress to illness and symptom reporting. *J. Psychosom. Res.* 41 115–128 10.1016/0022-3999(96)00119-5 8887825

[B12] Harris D. M. Guten S. (1979). Health protective behavior: an exploratory study. *J. Health Soc. Behav.* 20 17–29 10.2307/2136475 438490

[B13] Hill E. M. Maggi S. (2011). Emotional intelligence and smoking: protective and risk factors among Canadian young adults. *Pers. Individ. Dif.* 51 45–50 10.1016/j.paid.2011.03.008

[B14] Johnson S. J. Batey M. Holdsworth L. (2009). Personality and health: the mediating role of trait emotional intelligence and work locus of control. *Pers. Individ. Dif.* 47 470–475 10.1016/j.paid.2009.04.025

[B15] Jolic-Marjanovic Z. Altaras-Dimitrijevic A. (2014). Reliability, construct and criterion-related validity of the serbian adaptation of the trait emotional intelligence questionnaire (TEIQue). *Psihologija* 47 249–262 10.2298/PSI1402249J

[B16] Krick J. Sobal J. (1990). Relationships between health protective behaviors. *J. Community Health* 15 19–34 10.1007/BF01350183 2341603

[B17] Kun B. Demetrovics Z. (2010). Emotional intelligence and addictions: a systematic review. *Subst. Use Misuse* 45 1131–1160 10.3109/10826080903567855 20441455

[B18] Laborde S. Lautenbach F. Allen M. S. Herbert C. Achtzehn S. (2014). The role of trait emotional intelligence in emotion regulation and performance under pressure. *Pers. Individ. Dif.* 57 43–47 10.1016/j.paid.2013.09.013

[B19] Limonero J. T. Tomás-Sábado J. Fernández-Castro J. (2006). Perceived emotional intelligence and its relation to tobacco and cannabis use among university students. *Psicothema* 18(Suppl. 1) 95–100. 17295964

[B20] Martins A. Ramalho N. Morin E. (2010). A comprehensive meta-analysis of the relationship between emotional intelligence and health. *Pers. Individ. Dif.* 49 554–564 10.1016/j.paid.2010.05.029

[B21] Mavroveli S. Petrides K. V. Sangareau Y. Furnham A. (2009). Relating trait emotional intelligence to objective socioemotional outcomes in childhood. *Br. J. Educ. Psychol.* 79 259–272 10.1348/000709908X368848 18950549

[B22] Mayer J. Roberts R. D. Barsade S. G. (2008). Human abilities: trait EI. *Annu. Rev. Psychol.* 59 507–536 10.1146/annurev.psych.59.103006.093646 17937602

[B23] McHorney C. A. Ware J. E. Lu J. F. R. Sherbourne C. D. (1994). The MOS 36-item short-form health survey (SF-36): III. Tests of data quality, scaling assumptions and reliability across diverse patient groups. *Med. Care* 32 40–66 10.1097/00005650-199401000-00004 8277801

[B24] McHorney C. A. Ware J. E. Rogers W. Raczek A. Lu J. F. R. (1992). The validity and relative precision of MOS sort- and long-form health status scales and Dartmouth COOP charts: results from the medical outcomes study. *Med. Care* 30 253–265 10.1097/00005650-199205001-00025 1583937

[B25] Mikolajczak M. Luminet O. Leroy C. Roy E. (2007). Psychometric properties of the trait emotional intelligence questionnaire: factor structure, reliability, construct, and incremental validity in a French-speaking population. *J. Pers. Assess.* 88 338–353 10.1080/00223890701333431 17518555

[B26] Mikolajczak M. Luminet O. Menil C. (2006). Predicting resistance to stress: incremental validity of trait emotional intelligence over alexithymia and optimism. *Psicothema* 18 (Suppl. 1) 79–88. 17295962

[B27] Paek E. (2006). Religiosity and perceived emotional intelligence among christians. *Pers. Individ. Dif.* 41 479–490 10.1016/j.paid.2006.01.016

[B28] Peña-Sarrionandia A. Mikolajczak M. Gross J. J. (2015). Integrating emotion regulation and emotional intelligence traditions: a meta-analysis. *Front. Psychol.* 6:160 10.3389/fpsyg.2015.00160 PMC4338658 25759676

[B29] Pérez J. C. Petrides K. V. Furnham A. (2005). “Measuring trait emotional intelligence,” in *International Handbook of Emotional Intelligence* eds Schulze R. Roberts R. D. (Cambridge, MA: Hogrefe & Huber) 123–143.

[B30] Petrides K. V. (2009a). *Technical Manual for the Trait Emotional Intelligence Questionnaires (Teique)*. London: London Psychometric Laboratory.

[B31] Petrides K. V. (2009b). “Psychometric properties of the trait emotional intelligence questionnaire,” in *Advances in the Assessment of Emotional Intelligence: Theory, Research, And Applications* eds Stough C. Saklofske D. H. Parker J. D. (New York, NY: Springer) 85–101.

[B32] Petrides K. V. Furnham A. (2001). Trait emotional intelligence: psychometric investigation with reference to established trait taxonomies. *Eur. J. Pers.* 15 425–448 10.1002/per.416

[B33] Petrides K. V. Furnham A. (2003a). *Technical Manual of the Trait Emotional Intelligence Questionnaire (Teique)*. London: Institute of Education, University of London.

[B34] Petrides K. V. Furnham A. (2003b). Trait emotinal intelligence: behavioral validation in two studies of emotion recognition and reactivity to mood induction. *Eur. J. Pers.* 17 39–57 10.1002/per.466

[B35] Petrides K. V. Furnham A. Mavroveli S. (2007a). “Trait emotional intelligence. Moving forward in the field of EI,” in *Emotional Intelligence. Knowns and Unknowns (Series in Affective Science)* eds Matthews G. Zeidner M. Roberts R. (Oxford: Oxford University Press).

[B36] Petrides K. V. Pérez-González J. C. Furnham A. (2007b). On the criterion and incremental validity of trait emotional intelligence. *Cogn. Emot.* 21 26–55 10.1080/02699930601038912

[B37] Petrides K. V. Pita R. Kokkinaki F. (2007c). The location of trait emotional intelligence in personality factor space. *Br. J. Psychol.* 98 273–289 10.1348/000712606X120618 17456273

[B38] Saklofske D. H. Austin E. J. Galloway J. Davidson K. (2007). Individual difference correlates of health-related behaviors: preliminary evidence for links between emotional intelligence and coping. *Pers. Individ. Dif.* 42 491–502 10.1016/j.paid.2006.08.006

[B39] Saklofske D. H. Austin E. J. Minski P. S. (2003). Factor structure and validity of a trait emotional intelligence measure. *Pers. Individ. Dif.* 34 707–721 10.1016/S0191-8869(02)00056-9

[B40] Salovey P. Mayer J. D. (1990). Emotional intelligence. *Imagin. Cogn. Pers.* 9 185–211 10.2190/DUGG-P24E-52WK-6CDG

[B41] Salovey P. Mayer J. D. Goldman S. L. Turvey C. Palfai T. P. (1995). Emotional attention, clarity, and repair: exploring emotional intelligence using the trait meta-mood scale,” in *Emotion, Disclosure, and Health* ed. Pennebaker J. W. (Washington: American Psychological Association) 125–154.

[B42] Salovey P. Stroud L. R. Woolery A. Epel E. S. (2002). Perceived emotional intelligence, stress reactivity, and symptom reports: further explorations using the trait meta-mood scale. *Psychol. Health* 17 611–627 10.1080/08870440290025812

[B43] Siegling A. B. Saklofske D. H. Petrides K. V. (2014). “Measures of ability and trait emotional intelligence,” in *Measures of Personality and Social Psychological Constructs* eds Boyle G. J. Matthews G. Saklofske D. H. (San Diego: Elsevier/Academic Press) 381–414.

[B44] Tapp J. T. Goldenthal P. (1982). A factor analytic study of health habits. *Prev. Med.* 11 724–728 10.1016/0091-7435(82)90035-4 7163149

[B45] Tsaousis I. Nikolaou I. (2005). Exploring the relationship of emotional intelligence with physical and psychological health functioning. *Stress Health* 21 77–86 10.1002/smi.1042

[B46] Vickers R. R. Conway T. L. Hervig L. K. (1990). Demonstration of replicable dimensions of health behaviors. *Prev. Med.* 19 377–401 10.1016/0091-7435(90)90037-K 2399221

[B47] Ware J. E. Gandek B. (1998). Overview of the SF-36 health survey and the international quality of life assessment (IQOLA) project. *J. Clin. Epidemiol.* 51 903–912 10.1016/S0895-4356(98)00081-X 9817107

[B48] Ware J. E. Kosinski M. Bayliss M. S. McHorney C. A. Rogers W. H. Raczek A. (1995). Comparison of methods for the scoring and statistical analysis of SF-36 health profile and summary measures: summary of results from the medical outcomes study. *Med. Care* 33(Suppl. 4) AS264–AS279. 7723455

[B49] Ware J. E. Sherbourne C. D. (1992). The MOS 36-item short-form health survey (SF-36): I. conceptual framework and ítem selection. *Med. Care* 30 473–483 10.1097/00005650-199206000-00002 1593914

